# Clinical risk factors of stroke and major bleeding in patients with non-valvular atrial fibrillation under rivaroxaban: the EXPAND Study sub-analysis

**DOI:** 10.1007/s00380-019-01425-x

**Published:** 2019-05-24

**Authors:** Ichiro Sakuma, Shinichiro Uchiyama, Hirotsugu Atarashi, Hiroshi Inoue, Takanari Kitazono, Takeshi Yamashita, Wataru Shimizu, Takanori Ikeda, Masahiro Kamouchi, Koichi Kaikita, Koji Fukuda, Hideki Origasa, Hiroaki Shimokawa

**Affiliations:** 1 Cardiovascular Medicine, Hokko Memorial Clinic, Sapporo, Hokkaido Japan; 2 Center for Brain and Cerebral Vessels, Sanno Hospital and Sanno Medical Center, International University of Health and Welfare, 8-5-35 Akasaka, Minato-ku, Tokyo, Japan; 3 Minamihachioji Hospital, 3-18-12, Koyasu-cho, Hachioji, Tokyo Japan; 4 Saiseikai Toyama Hospital, 33-1, Kusunoki, Toyama, Toyama Japan; 5 Department of Medicine and Clinical Science, Graduate School of Medical Sciences, Kyushu University, 3-1-1, Maidashi, Higashi-ku, Fukuoka, Fukuoka Japan; 6 Cardiovascular Institute Hospital, 3-2-19 Nishiazabu, Minato-Ku, Tokyo, Japan; 7 Department of Cardiovascular Medicine, Graduate School of Medicine, Nippon Medical School, 1-1-5, Bunkyo-ku, Sendagi, Tokyo Japan; 8 Department of Cardiovascular Medicine, Faculty of Medicine, Toho University, 5-21-16, Omorinishi, Ota-ku, Tokyo, Japan; 9 Department of Health Care Administration and Management, Center for Cohort Study, Kyushu University Graduate School of Medical Sciences, 3-1-1, Maidashi, Higashi-ku, Fukuoka, Fukuoka Japan; 10 Department of Cardiovascular Medicine, Graduate School of Medical Sciences, Kumamoto University, 2-39-1, Kurokami Chuo-ku, Kumamoto, Kumamoto Japan; 11 Division of Heart Rhythm, International University of Health and Welfare Hospital, International University of Health and Welfare, 537-3, Iguchi, Nasushiobara, Tochigi Japan; 12 Division of Biostatistics and Clinical Epidemiology, University of Toyama Graduate School of Medicine, 2630 Sugitani, Toyama, Toyama Japan; 13 Department of Cardiovascular Medicine, Tohoku University Graduate School of Medicine, 1-1, Seiryomachi, Aoba-ku, Sendai, 980-8574 Miyagi Japan

**Keywords:** CHADS_2_, CHA_2_DS_2_-VASc, HAS-BLED, Non-valvular atrial fibrillation, Rivaroxaban, Risk factor

## Abstract

For Japanese patients with non-valvular atrial fibrillation (NVAF), the risk of stroke and major bleeding events was assessed by using the CHADS_2,_ CHA_2_DS_2_-VASc, and HAS-BLED scores. The risk factors for embolism and major bleeding under DOAC may be different from current reports. We analyzed the data set of the EXPAND Study to determine the risk factors for events among Japanese NVAF patients in the era of direct oral anticoagulant. Using the data of EXPAND Study, the validity for predictability of the CHADS_2_, CHA_2_DS_2_-VASc, and HAS-BLED scores was identified using the receiver operating characteristic curve analysis. Multivariate analysis was performed with the Cox proportional hazard model to determine the independent risk factors for stroke/systemic embolism and major bleeding among NVAF patients receiving rivaroxaban. Explanatory variables were selected based on the univariate analysis. A total of 7141 patients (mean age 71.6 ± 9.4 years, women 32.3%, and rivaroxaban 15 mg per day 56.5%) were included. Incidence rates of stroke/systemic embolism and major bleeding were 1.0%/year and 1.2%/year, respectively. The multivariate analysis revealed that only history of stroke was associated with stroke/systemic embolism (hazard ratio 3.4, 95% confidence interval 2.5-4.7, *p *< 0.0001). By contrast, age (1.7, 1.1–2.6, *p *= 0.0263), creatinine clearance (CrCl) 30–49 mL/min (1.6, 1.2-2.2, *p *= 0.0011), liver dysfunction (1.7, 1.1–2.8, *p *= 0.0320), history/disposition of bleeding (1.8, 1.0–3.0, *p *= 0.0348), and concomitant use of antiplatelet agents (1.6, 1.2–2.3, *p *= 0.0030) were associated with major bleeding. This sub-analysis showed that some components of the HAS-BLED score were independently associated with major bleeding in Japanese NVAF patients receiving anticoagulation therapy by rivaroxaban. Additionally, CrCl value of 30–49 mL/min was an independent predictor of major bleeding in patients receiving rivaroxaban.

## Introduction

Non-valvular atrial fibrillation (NVAF) is a common disease in the cardiovascular field, but is also a type of arrhythmia often observed in other medical fields. It can occur without any clear underlying diseases, with the prevalence increasing with aging. The overall prevalence of NVAF in Japan is 0.56%, estimated using the data from periodical medical checkups of patients aged ≥ 40 years [1, 2]. Of the patients, 85% were older than 60 years and 25% were older than ≥ 80 years, leading to an overall estimated prevalence of 1.09% in 2050 [1, 2].

For evaluation of thromboembolic risks in patients with NVAF, CHADS_2_ (congestive heart failure [CHF], hypertension [HT], age ≥ 75 years, diabetes mellitus [DM], previous stroke/transient ischemic attack [TIA]) score has been used and validated as optimal in Japanese patients [3–5]. Among Japanese NVAF patients without anticoagulation, the incidence rate of ischemic stroke has been reported to be 13.3 per 1000 person-years by the pooled analysis of data from J-RHYTHM Registry, Fushimi AF Registry, and Shinken Database [4]. CHA_2_DS_2_-VASc (CHF, HT, age ≥ 75 years, DM, previous stroke/TIA, vascular disease, age 65–74 years, female; age ≥ 75 years and previous stroke carry doubled risk weight) score was introduced in Europe to extract truly low-risk patients from patients with CHADS_2_ score of 0 and 1 [6, 7]. In the above-mentioned pooled analysis in Japanese patients without anticoagulants, an increase in incidence of ischemic stroke was observed in Japanese patients with CHA_2_DS_2_-VASc scores ≥ 2 [4]. Furthermore, although other Japanese data validated the usefulness of CHA_2_DS_2_-VASc score, its full applicability remains uncertain in the era of direct oral anticoagulants (DOACs) [8–11]. Risk stratification schemes used to predict bleeding events during treatment with anticoagulants include HAS-BLED, ORBIT, and ATRIA; however, HAS-BLED score has been used together with CHA_2_DS_2_-VASc score in the guidelines of the European Society of Cardiology [7, 12, 13]. The usefulness of HAS-BLED score was also validated in Japanese patients [10, 14].

The EXPAND Study is a prospective observational cohort study in patients with NVAF who were treated with Japan-specific dosages of rivaroxaban to determine the efficacy and safety in the real-world clinical setting [15, 16]. The risk factors for embolism and major bleeding under DOAC may be different from current reports. In this sub-analysis, we determined the relationship of each component of CHADS_2_, CHA_2_DS_2_-VASc, and HAS-BLED scores with thromboembolic and bleeding events among Japanese NVAF patients receiving Japan-specific dosage of rivaroxaban, and validated the predictability of those scores.

## Methods

### Study design and outcome

The EXPAND Study (Evaluation of the effectiveness and safety of Xa inhibitor for the Prevention of stroke And systemic embolism [SE] in a Nationwide cohort of Japanese patients Diagnosed as NVAF) is an investigator-initiated multicenter registry conducted from November 2012 to March 2016 to evaluate the efficacy and safety of rivaroxaban for prevention of stroke/SE in Japanese NVAF patients in the real-world clinical practice as reported elsewhere [15, 16]. Briefly, 7141 patients with NVAF aged ≥ 20 years were included from 684 medical institutes (mean age 71.6 ± 9.4 years, women 32.3%). They were followed for a mean duration of 2.5 years. The endpoints were stroke/SE and major bleeding events (defined as International Society on Thrombosis and Haemostasis [ISTH] major bleeding criteria) [15, 16]. The incidence rates for stroke/SE and ISTH major bleeding were 1.0%/year (176 events) and 1.2%/year (215 events), respectively [16].

The present study was conducted in accordance with the Declaration of Helsinki; the Ethical Guidelines for Clinical Studies by the Japanese Ministry of Health, Labour and Welfare; and all applicable laws and regulations in Japan. The protocol was reviewed and approved by the institutional review boards and/or ethics committees in all the participating institutes. All patients provided written informed consent before enrollment in this study. This study is registered with ClinicalTrials.gov (NCT02147444) and the University Hospital Medical Information Network clinical trials registry (UMIN000009376).

### Statistical analysis

The incidence rates of outcome events (%/year) from time of starting rivaroxaban to the initial onset of events were compared according to patient characteristics and medical history. The validity for prediction ability of the CHADS_2_, CHA_2_DS_2_-VASc and HAS-BLED scores was identified using the receiver operating characteristic (ROC) curve analysis. Hazard ratio (HR), 95% confidence interval (CI), and *p* value for thromboembolic and bleeding events were estimated using Cox proportional hazards model. Multivariate analysis was conducted below model. Components of CHADS_2_ and CHA_2_DS_2_-VASc scores were selected for thromboembolic events, whereas components of HAS-BLED score were selected for major bleeding. Labile prothrombin time international normalized ratio was handled as “data not available” for HAS-BLED score. The factors selected showing a significant difference (*p* < 0.05) in the univariate analysis. P values of < 0.05 were considered to be statistically significant. All statistical analyses were conducted using the SAS software (SAS for Windows Release ver. 9.2 or later, SAS Institute Inc.).

## Results

### Patient characteristics

Baseline characteristics of the patients are listed in Table 1. Among 7141 patients enrolled, approximately 80% aged ≥ 65 years, 40% aged ≥ 75 years, and 70% had HT. Twenty percent of patients had prior history of ischemic stroke. A total of 972 patients (13.6%) were at lower risk for thromboembolism, with CHA_2_DS_2_-VASc scores of 0 and 1. Among patients complicated with HT and DM, 4675 patients (94.5%) and 1118 patients (91.3%) received antihypertensive and antidiabetic medications at baseline of this study, respectively.

**Table 1:** Patient characteristics according to CHA_2_DS_2_-VASc score

	Overall	CHA_2_DS_2_-VASc score				
		0	1	2	3–5	6–9
No. of patients (%)	7141 (100.0)	222 (3.1)	750 (10.5)	1317 (18.4)	4087 (57.2)	765 (10.7)
Sex (female)	2303 (32.3)	0 (0.0)	64 (8.5)	250 (19.0)	1551 (37.9)	438 (57.3)
Age (years)						
< 65	1436 (20.1)	222 (100.0)	481 (64.1)	400 (30.4)	327 (8.0)	6 (0.8)
65–74	2786 (39.0)	0 (0.0)	269 (35.9)	781 (59.3)	1646 (40.3)	90 (11.8)
≥ 65	5705 (79.9)	0 (0.0)	269 (35.9)	917 (69.6)	3760 (92.0)	759 (99.2)
≥ 75	2919 (40.9)	0 (0.0)	0 (0.0)	136 (10.3)	2114 (51.7)	669 (87.5)
Body weight (kg)						
≥ 60	4047 (59.0)	186 (89.0)	569 (79.9)	887 (70.8)	2113 (53.6)	292 (39.2)
50–59	1855 (27.0)	22 (10.5)	121 (17.0)	284 (22.7)	1177 (29.9)	251 (33.7)
< 50	956 (13.9)	1 (0.5)	22 (3.1)	82 (6.5)	650 (16.5)	201 (27.0)
SBP (mmHg) ≥ 160	310 (4.6)	3 (1.5)	14 (2.0)	41 (3.3)	203 (5.2)	49 (6.7)
CrCl (mL/min)						
≥ 50	5326 (78.3)	204 (100.0)	692 (98.0)	1153 (93.0)	2874 (73.4)	403 (54.3)
30–49	1347 (19.8)	0 (0.0)	14 (2.0)	83 (6.7)	952 (24.3)	298 (40.2)
< 30	133 (2.0)	0 (0.0)	0 (0.0)	4 (0.3)	88 (2.2)	41 (5.5)
CHADS_2_ score						
< 2	2667 (37.3)	222 (100.0)	750 (100.0)	1080 (82.0)	615 (15.0)	0 (0.0)
2	2064 (28.9)	0 (0.0)	0 (0.0)	237 (18.0)	1827 (44.7)	0 (0.0)
≥ 3	2410 (33.7)	0 (0.0)	0 (0.0)	0 (0.0)	1645 (40.2)	765 (100.0)
HAS-BLED score						
< 3	5928 (88.8)	191 (99.5)	673 (98.5)	1163 (96.0)	3421 (88.7)	480 (65.8)
≥ 3	746 (11.2)	1 (0.5)	10 (1.5)	49 (4.0)	436 (11.3)	250 (34.2)
Comorbidity						
CHF	1864 (26.1)	0 (0.0)	53 (7.1)	180 (13.7)	1217 (29.8)	414 (54.1)
Hypertension	5065 (70.9)	0 (0.0)	316 (42.1)	810 (61.5)	3229 (79.0)	710 (92.8)
Angina pectoris	833 (11.7)	0 (0.0)	2 (0.3)	48 (3.6)	538 (13.2)	245 (32.0)
Diabetes mellitus	1737 (24.3)	0 (0.0)	46 (6.1)	185 (14.0)	1099 (26.9)	407 (53.2)
PAD	187 (2.6)	0 (0.0)	0 (0.0)	12 (0.9)	112 (2.7)	63 (8.2)
Aortic aneurysm	98 (1.4)	0 (0.0)	0 (0.0)	6 (0.5)	67 (1.6)	25 (3.3)
Deep vein thrombosis	37 (0.5)	1 (0.5)	1 (0.1)	2 (0.2)	20 (0.5)	13 (1.7)
Pulmonary embolism	18 (0.3)	0 (0.0)	1 (0.1)	2 (0.2)	12 (0.3)	3 (0.4)
Dyslipidemia	2995 (41.9)	62 (27.9)	228 (30.4)	516 (39.2)	1788 (43.7)	401 (52.4)
Liver dysfunction	413 (5.8)	13 (5.9)	41 (5.5)	91 (6.9)	240 (5.9)	28 (3.7)
Renal dysfunction	7 (0.1)	0 (0.0)	0 (0.0)	1 (0.1)	5 (0.1)	1 (0.1)
Medical history						
Ischemic stroke	1440 (20.2)	0 (0.0)	0 (0.0)	39 (3.0)	860 (21.0)	541 (70.7)
Hemorrhagic stroke	135 (1.9)	1 (0.5)	9 (1.2)	15 (1.1)	89 (2.2)	21 (2.7)
Transient ischemic attack	219 (3.1)	0 (0.0)	0 (0.0)	6 (0.5)	132 (3.2)	81 (10.6)
Systemic embolism	59 (0.8)	0 (0.0)	6 (0.8)	6 (0.5)	33 (0.8)	14 (1.8)
Myocardial infarction	298 (4.2)	0 (0.0)	0 (0.0)	9 (0.7)	207 (5.1)	82 (10.7)
Malignant tumor	654 (9.2)	13 (5.9)	59 (7.9)	116 (8.8)	388 (9.5)	78 (10.2)
Bleeding/disposition of bleeding	292 (4.1)	5 (2.3)	21 (2.8)	48 (3.6)	179 (4.4)	39 (5.1)
Rivaroxaban dosage 15 mg/day	4036 (56.5)	198 (89.2)	639 (85.2)	950 (72.1)	2024 (49.5)	225 (29.4)
Amount of drinking (unit/week)						
Nondrinker	3798 (53.2)	65 (29.3)	247 (32.9)	518 (39.3)	2410 (59.0)	558 (72.9)
< 8	2402 (33.6)	112 (50.5)	303 (40.4)	556 (42.2)	1261 (30.9)	170 (22.2)
≥ 8	941 (13.2)	45 (20.3)	200 (26.7)	243 (18.5)	416 (10.2)	37 (4.8)
History of smoking						
Nonsmoker	4318 (60.5)	115 (51.8)	371 (49.5)	706 (53.6)	2568 (62.8)	558 (72.9)
In the past	2097 (29.4)	63 (28.4)	242 (32.3)	428 (32.5)	1199 (29.3)	165 (21.6)
Current	726 (10.2)	44 (19.8)	137 (18.3)	183 (13.9)	320 (7.8)	42 (5.5)
Type of AF						
Non-PAF^a^	3940 (55.2)	119 (53.6)	392 (52.3)	659 (50.0)	2296 (56.2)	474 (62.0)
Using concomitant anti-platelets	1029 (14.4)	2 (0.9)	23 (3.1)	92 (7.0)	669 (16.4)	243 (31.8)
Using concomitant NSAIDs	165 (2.3)	4 (1.8)	12 (1.6)	22 (1.7)	96 (2.3)	31 (4.1)

*SBP* systolic blood pressure, *CrCl* creatinine clearance, *CHF* congestive heart failure, *PAD* peripheral arterial disease, *AF* atrial fibrillation, *PAF* paroxysmal atrial fibrillation, *NSAIDs* non-steroidal anti-inflammatory drugs

^a^Persistent and permanent atrial fibrillation

### Outcome of sub-group analysis

**Fig. 1:**
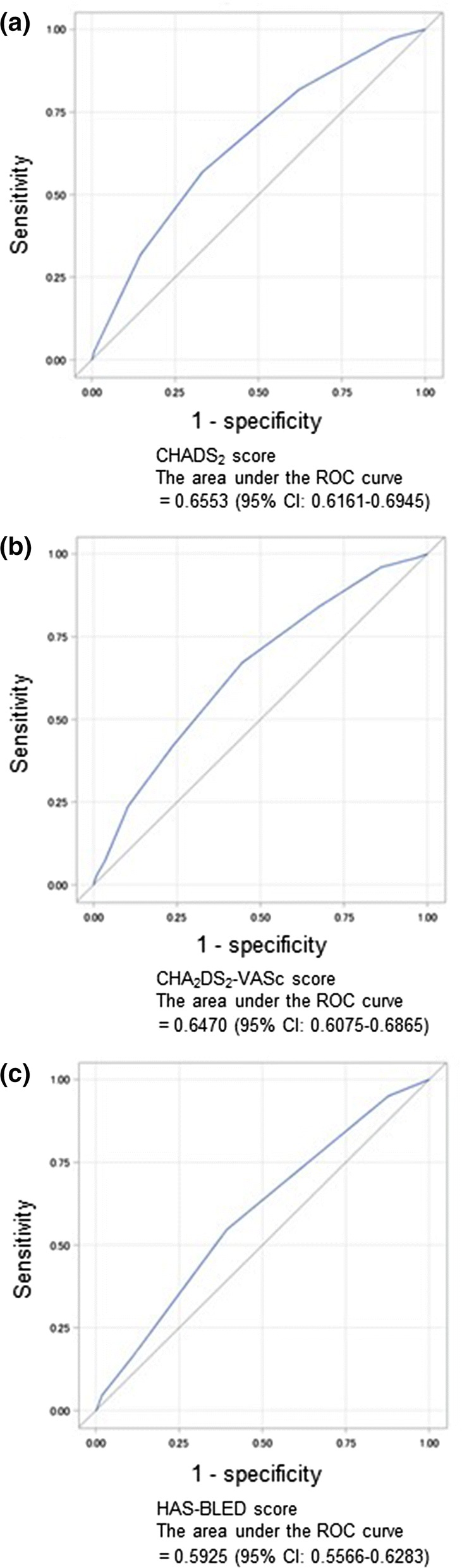
Predictability of the (**a**) CHADS_2_ and (**b**) CHA_2_DS_2_-VASc scores for stroke/systemic embolism, and (**c**) HAS-BLED score for major bleeding analyzed by the receiver operating characteristic curve

The results of validity for predictability of the scores are shown in Fig. 1. The area under the ROC curve and score of cut-off in CHADS_2_, CHA_2_DS_2_-VASc, and HAS-BLED were 0.6553 (95% CI 0.6161–0.6945) and 3, 0.6470 (95% CI 0.6075–0.6865) and 4, and 0.5925 (95% CI 0.5566–0.6283) and 2, respectively (Fig. 1). In any scores, the area under the ROC curve analysis showed low accuracy of predictability for risk of thromboembolic and bleeding events.

**Fig. 2:**
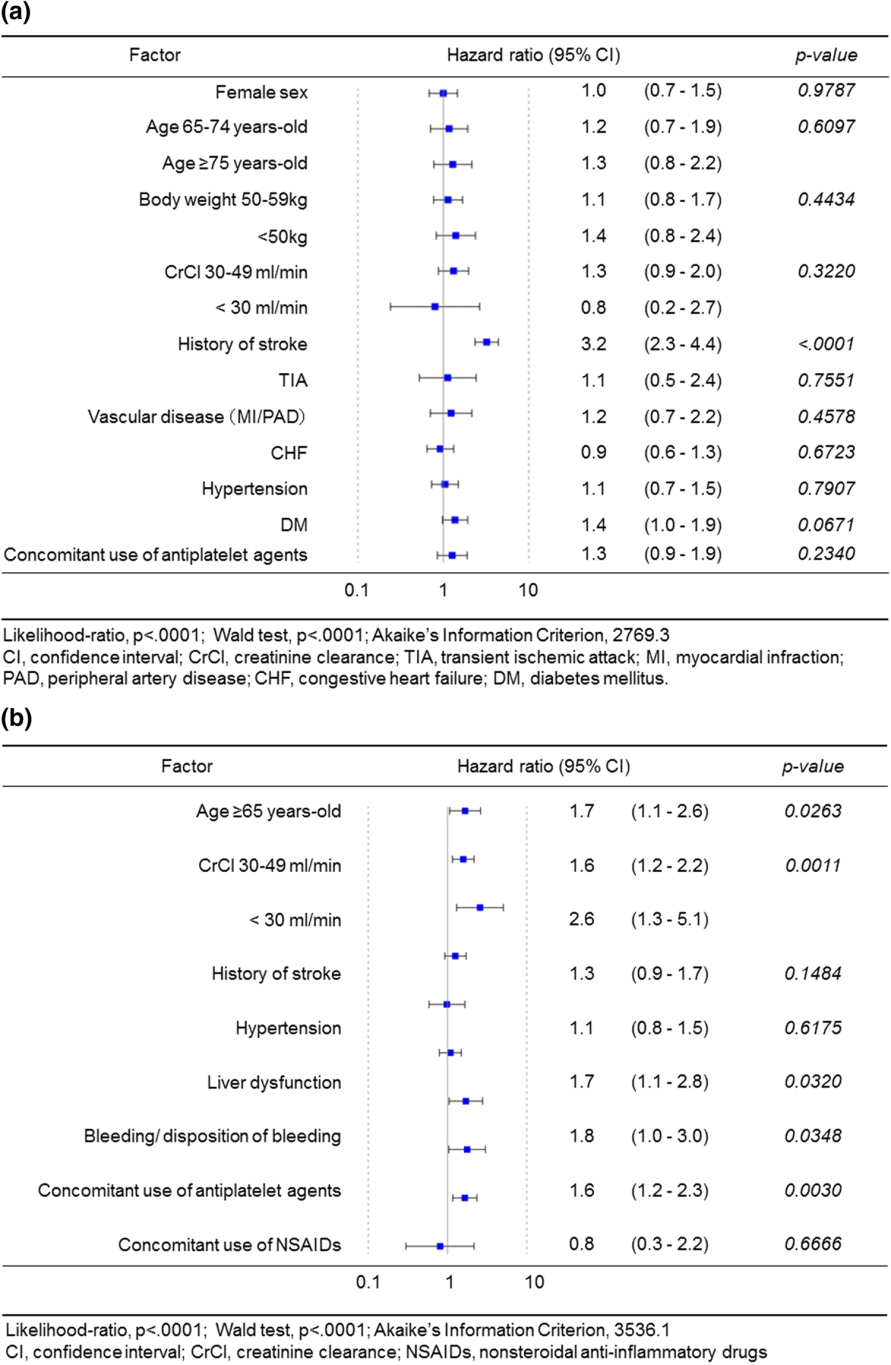
Multivariate analysis by Cox proportional hazard model for risk of (a) stroke/systemic embolism and (b) major bleeding

The results of the incidence rates and univariate analysis using Cox proportional hazards model are shown in Tables 2 and 3. The results of the multivariate analysis using the Cox proportional hazards model are summarized in Fig. 2. The CHADS_2_, CHA_2_DS_2_-VASc and HAS-BLED scores showed a significant difference in univariate analysis, although those factors were not included for multivariate analysis due to a high correlation coefficient. Only prior history of stroke (ischemic/hemorrhagic) was associated with stroke/SE (HR 3.2, 95% CI 2.3–4.4, *p* < 0.0001), but other components of CHA_2_DS_2_-VASc score were not (Fig. 2a). By contrast, several components of HAS-BLED score were associated with major bleeding (Fig. 2b). Among them were age ≥ 65 years, liver dysfunction, history/disposition of bleeding, and concomitant use of antiplatelet drugs. Additionally, CrCl of 30–49 mL/min and < 30 mL/min was independently associated with major bleeding. Renal dysfunction was not included in both univariate and multivariate analysis due to the small number of patients (7 patients) with this diagnosis and events (stroke/SE; 0 event, major bleeding; 3 events) (Table 1).

**Table 2:** Incidence rate and univariate analysis by Cox proportional hazards analysis of stroke/systemic embolism

	No. of events (%/year)	HR	95% CI	*p* value
Overall	176 (1.0)			
Sex				
Male	115 (1.0)	Reference		0.5283
Female	61 (1.1)	1.1	0.8, 1.5	
Age class 1 (years-old)				
< 65	23 (0.7)	Reference		0.0176
≥ 65	153 (1.1)	1.7	1.1, 2.6	
Age class 2 (years-old)				
< 65	23 (0.7)	Reference		0.0022
65–74	60 (0.9)	1.3	0.8, 2.2	
≥ 75	93 (1.3)	2.0	1.3, 3.2	
Body weight (kg)				
≥ 60	84 (0.8)	Reference		0.0250
50–59	50 (1.1)	1.3	0.9, 1.9	
< 50	33 (1.4)	1.7	1.1, 2.6	
Systolic blood pressure (mmHg)				
< 160	156 (1.0)	Reference		0.0928
≥ 160	12 (1.6)	1.6	0.9, 3.0	
CrCl (mL/min)				
≥ 50	112 (0.9)	Reference		0.0026
30–49	49 (1.5)	1.8	1.3, 2.5	
< 30	3 (1.0)	1.2	0.4, 3.7	
Comorbidity^a^				
Congestive heart failure				
–	129 (1.0)	1.0	0.7, 1.4	0.8550
+	47 (1.0)			
Hypertension				
–	44 (0.9)	1.2	0.9, 1.7	0.2768
+	132 (1.1)			
Angina pectoris				
–	157 (1.0)	0.9	0.6, 1.5	0.7193
+	19 (0.9)			
Diabetes mellitus				
–	121 (0.9)	1.4	1.1, 2.0	0.0227
+	55 (1.3)			
Aortic aneurysm				
–	174 (1.0)	0.8	0.2, 3.3	0.7916
+	2 (0.8)			
Deep vein thrombosis				
–	175 (1.0)	1.0	0.2, 7.4	0.9718
+	1 (1.0)			
Pulmonary embolism				
–	175 (1.0)	2.3	0.3, 16.2	0.4012
+	1 (2.2)			
Dyslipidemia				
–	105 (1.0)	0.9	0.7, 1.3	0.6278
+	71 (1.0)			
Liver dysfunction				
–	167 (1.0)	0.9	0.5, 1.7	0.6999
+	9 (0.9)			
Renal dysfunction				
–	176 (1.0)	< 0.001	< 0.001	0.6995
+	0 (0.0)			
Medical history^a^				
Stroke (ischemic/hemorrhagic)				
–	91 (0.7)	Reference		< 0.0001
+	85 (2.3)	3.6	2.7, 4.8	
Transient ischemic attack				
–	169 (0.9)	Reference		0.4721
+	7 (1.3)	1.3	0.6, 2.8	
Systemic embolism				
–	174 (1.0)	Reference		0.6643
+	2 (1.4)	1.4	0.3, 5.5	
Vascular disease (MI/PAD)				
–	158 (1.0)	Reference		0.0352
+	18 (1.6)	1.7	1.0, 2.7	
Malignant tumor				
–	159 (1.0)	Reference		0.7657
+	17 (1.1)	1.1	0.7, 1.8	
Bleeding/disposition of bleeding				
–	169 (1.0)	Reference		0.9900
+	7 (1.0)	1.0	0.5, 2.1	
Rivaroxaban dosage				
15 mg/day	87 (0.9)	Reference		0.0658
10 mg/day	89 (1.2)	1.3	1.0, 1.8	
Amount of drinking (unit/week)				
No	105 (1.1)	Reference		0.1202
< 8	47 (0.8)	0.7	0.5, 1.0	
≥8	24 (1.0)	0.9	0.6, 1.4	
History of smoking				
No	99 (0.9)	Reference		0.2940
In the past	54 (1.1)	1.1	0.8, 1.6	
Current	23 (1.3)	1.4	0.9, 2.2	
Type of AF				
PAF	67 (0.9)	Reference		0.0799
Non-PAF^b^	109 (1.1)	1.3	1.0, 1.8	
Using concomitant anti-platelets^a^				
–	138 (0.9)	Reference		0.0046
+	38 (1.5)	1.7	1.2, 2.4	
Using concomitant NSAIDs^a^				
–	172 (1.0)	Reference		*0.9525*
+	4 (1.0)	1.0	0.4, 2.8	
CHADS_2_ score				
< 3	76 (0.7)	Reference		< *0.0001*
≥ 3	100 (1.7)	2.7	2.0, 3.6	
CHA_2_DS_2_-VASc score				
< 4	58 (0.6)	Reference		< *0.0001*
≥ 4	118 (1.5)	2.5	1.9, 3.5	
HAS-BLED score				
< 2	60 (0.6)	Reference		< *0.0001*
≥ 2	106 (1.7)	2.8	2.0, 3.8	< *0.0001*

*HR* hazard ratio, *CI* confidence interval, *CrCl* creatinine clearance, *MI* myocardial infarction, *PAD* peripheral arterial disease, *AF* atrial fibrillation, *PAF* paroxysmal atrial fibrillation, *NSAIDs* non-steroidal anti-inflammatory drugs

*P* values were determined by log-rank test

^a^Reference; without factor

^b^Persistent and permanent atrial fibrillation

**Table 3:** Incidence rate and univariate analysis by Cox proportional hazards analysis of ISTH major bleeding

	ISTH major bleeding			
	No. of events (%/year)	HR	95% CI	*p* value
Overall	176 (1.0)			
Sex				
Male	115 (1.0)	Reference		*0.8223*
Female	61 (1.1)	1.0	0.7, 1.3	
Age class 1 (years-old)				
< 65	26 (1.8)	Reference		*0.0027*
< 65	189 (3.3)	1.9	1.2, 2.8	
Age class 2 (years-old)				
< 65	23 (0.7)	Reference		<*.0001*
65–74	60 (0.9)	1.3	0.8, 2.1	
≥ 75	93 (1.3)	2.4	1.6, 3.6	
Body weight (kg)				
≥ 60	84 (0.8)	Reference		*0.7249*
50–59	50 (1.1)	0.9	0.7, 1.3	
< 50	33 (1.4)	1.1	0.7, 1.6	
Systolic blood pressure (mmHg)				
< 160	156 (1.0)	Reference		*0.2772*
≥ 160	12 (1.6)	0.6	0.3, 1.4	
CrCl (mL/min)				
≥ 50	112 (0.9)	Reference		< *0.0001*
30–49	49 (1.5)	1.8	1.3, 2.4	
< 30	3 (1.0)	2.9	1.5, 5.6	
Comorbidity^a^				
CHF				
–	149 (1.2)	Reference		*0.1140*
+	66 (1.4)	1.3	1.0, 1.7	
Hypertension				
–	53 (1.1)	Reference		*0.1770*
+	162 (1.3)	1.2	0.9, 1.7	
Angina pectoris				
–	181 (1.2)	Reference		*0.0543*
+	34 (1.7)	1.4	0.9, 2.1	
Diabetes mellitus				
–	159 (1.2)	Reference		*0.4925*
+	56 (1.3)	1.1	0.8, 1.5	
Aortic aneurysm				
–	210 (1.2)	Reference		*0.2144*
+	5 (2.1)	1.7	0.7, 4.2	
DVT				
–	212 (1.2)	Reference		*0.0823*
+	3 (3.1)	2.6	0.9, 8.3	
PE				
–	214 (1.2)			*0.5833*
+	1 (2.2)	1.7	0.2, 12.3	
Dyslipidemia				
–	125 (1.2)			*0.9155*
+	90 (1.2)	1.0	0.8, 1.3	
Liver dysfunction				
–	195 (1.2)			*0.0252*
+	20 (2.0)	1.7	1.1, 2.7	
Renal dysfunction				
–	215 (1.2)			*0.6682*
+	0 (0.0)	< 0.001	< 0.001, > 999.9	
Medical history				
Stroke (ischemic/hemorrhagic)				
–	154 (1.1)	Reference		*0.0065*
+	61 (1.7)	1.5	1.1, 2.0	
Transient ischemic attack				
–	208 (1.2)	Reference		*0.8415*
+	7 (1.3)	1.1	0.5, 2.3	
Systemic embolism				
–	215 (1.2)	Reference		*0.1736*
+	0 (0.0)	< 0.001	< 0.001, > 999.9	
Vascular disease (MI/PAD)				
–	196 (1.2)	Reference		*0.1345*
+	19 (1.7)	1.4	0.9, 2.3	
Malignant tumor				
–	189 (1.2)	Reference		*0.1145*
+	26 (1.7)	1.4	0.9, 2.1	
Bleeding/disposition of bleeding				
–	200 (1.2)	Reference		*0.0240*
+	15 (2.1)	1.8	1.1, 3.1	
Rivaroxaban dosage				
15 mg/day	108 (1.1)	Reference		*0.0635*
10 mg/day	107 (1.4)	1.3	1.0, 1.7	
Amount of drinking (unit/week)				
No	128 (1.4)	Reference		*0.0649*
< 8	57 (1.0)	0.7	0.5, 1.0	
≥ 8	30 (1.3)	0.9	0.6, 1.4	
History of smoking				
No	126 (1.2)	Reference		*0.4144*
In the past	71 (1.4)	1.2	0.9, 1.6	
Current	18 (1.0)	0.9	0.5, 1.4	
Type of AF				
PAF	89 (1.1)	Reference		*0.3453*
Non-PAF^b^	126 (1.3)	1.1	0.9, 1.5	
Using concomitant anti-platelets^a^				
–	166 (1.1)	Reference		*0.0003*
+	49 (2.0)	1.8	1.3, 2.5	
Using concomitant NSAIDs^a^				
–	211 (1.2)	Reference		*0.7265*
+	4 (1.0)	0.8	0.3, 2.3	
CHADS_2_ score				
< 3	121 (1.0)	Reference		*0.0009*
≥ 3	94 (1.6)	1.6	1.2, 2.1	
CHA_2_DS_2_-VASc score				
< 4	92 (1.0)	Reference		*0.0001*
≥ 4	123 (1.6)	1.7	1.3, 2.2	
HAS-BLED score				
< 2	92 (0.9)	Reference		< *0.0001*
≥ 2	111 (1.7)	1.9	1.4, 2.5	

*HR* hazard ratio, *CI* confidence interval, *ISTH* International Society on Thrombosis and Haemostasis, *CrCl* creatinine clearance, *MI* myocardial infarction, *PAD* peripheral arterial disease, *AF* atrial fibrillation, *PAF* paroxysmal atrial fibrillation, *NSAIDs* non-steroidal anti-inflammatory drugs

*P* values were determined by log-rank test

^a^Reference; without factor

^b^Persistent and permanent atrial fibrillation

## Discussion

The major findings of the present sub-analysis of EXPAND Study are as follows. First, the CHADS_2_, CHA_2_DS_2_-VASc, and HAS-BLED scores were of low accuracy for assessment of thromboembolic and bleeding events verifying by ROC curve analysis. However, the incidence rate in patients with high score was significantly increased compared with those with low score. Second, among Japanese NVAF patients receiving Japan-specific dosages of rivaroxaban, some components of HAS-BLED score were independently associated with major bleeding. Among them were age, liver dysfunction, history/disposition of bleeding, age ≥ 65 years, and concomitant use of antiplatelet agents. Additionally, lower values of creatinine clearance (CrCl) were associated with major bleeding. Third, only prior history of stroke emerged as an independent predictor of stroke/SE, but other components of CHA_2_DS_2_-VASc score did not so.

### Predictors of major bleeding

Several components of HAS-BLED score were associated with major bleeding as expected. In the present sub-analysis, number of patients clinically having renal dysfunction was small; therefore, we included CrCl values as a possible explanatory variable for multivariate analysis instead of renal dysfunction. Lower CrCl values were associated with major bleeding as expected from the ABC-Bleeding score [17], although HAS-BLED score was superior in evaluating the risk of developing a serious bleeding event for a long time period [18]. In J-RHYTHM Registry, CrCl values < 50 mL/min were associated with major bleeding in univariate analysis; however, they were not so in multivariate analysis [19].

Since a substantial portion of DOAC is excreted through the kidney, CrCl values could be closely associated with bleeding events. However, the relation between CrCl values and rivaroxaban dosage was not determined thoroughly in this sub-analysis, and further investigation should be conducted. The present study showed that patient with off-label dosage of rivaroxaban has approximately 24% and 2% in under-dosage and over-dosage, respectively. We plan to clarify the relationship between renal function and dosage of rivaroxaban in the ongoing exploratory analyses of our study.

### Predictors of thromboembolic events

Multivariate analysis revealed that the history of stroke alone was associated with stroke/SE. In the pooled analysis of data from J-RHYTHM Registry, Fushimi AF Registry, and Shinken Database [4], age ≥ 75 years, HT and history of stroke/TIA emerged as independent predictor of ischemic stroke for Japanese patients with NVAF not using anticoagulants. Furthermore, the Fushimi AF Registry reported that not only age and history of stroke, but also unstable heart failure (HF) (within 30 days after hospitalization due to HF), type B natriuretic peptide (BNP) level, or N-terminal fragment of the prohormone B natriuretic peptide (NT-pro BNP) level were independent risk factors [20].

In the main analysis of EXPAND Study [16], the incidence rate of stroke/SE increased along with an increase in CHA_2_DS_2_-VASc score. However, the incidence rate of stroke/SE was too low to determine impact of each component on incident stroke/SE in patients receiving rivaroxaban. The incidence of stroke in Japanese patients has been decreasing because of appropriate management of salt intake and blood pressure (BP), as well as decreased smoking rate [21]. In the present study, the proportion of patients complicated with HT using antihypertensive medications was 94.5%, which is higher than that of general Japanese patients with HT aged 60–69 years (65.6%) and aged 70–79 years (80.8%) in 2010 [22]. The patients complicated with HT may be the not-at-risk population for stroke if they undergo optimal antihypertensive treatment [22]. A sub-analysis of the J-RHYTHM Registry [23] clearly indicated BP control was closely associated with thromboembolic events as well as bleeding events. Although the proportion of patients with baseline systolic BP of ≥ 160 mmHg was quite low, i.e., 4.6%, information for BP control was not collected during the follow-up period in the present study. The patients of this study were treated with rivaroxaban, mostly treated by a cardiologist or a physician who was interested in anticoagulation. Therefore, it is possible that the patient’s condition was being well and the incidence rate of thromboembolic events were lower than patients who were not so.

### Predictability of thromboembolic and bleeding events

The present sub-analysis showed that CHADS_2_, CHA_2_DS_2_-VASc, and HAS-BLED scores had low accuracy of predictability for thromboembolic and bleeding events in patients treated with rivaroxaban under daily clinical practice verifying by ROC curve analysis. However, the other analyses of this study showed that the incidence rates increased with the higher score [16], and the rate was higher in the patients with high score than in those with low score (Tables 2 and 3). Although the ROC curve analysis did not show predictability for thromboembolism and bleeding events, the scores have enough predictability for those in NVAF patients treated with rivaroxaban. Additional clinical trials may be needed to develop highly predictive tool for thromboembolic and bleeding events in NVAF patients treated with DOACs.

### Study limitations

The present study had several limitations [15, 16]. First, several biases may have affected the present results. For instance, rivaroxaban-naïve as well as rivaroxaban-experienced patients were included. Second, patients who were switched to other oral anticoagulants from rivaroxaban and did not continue anticoagulation were included. Third, only clinical risk factors were selected for multivariate analysis, and laboratory data except for CrCl such as NT-pro BNP, growth differentiation factor-15, high-sensitivity cardiac troponin T, prothrombin time and hemoglobin [7, 24, 25] were not included. Finally, detailed information for management of comorbidities such as HF, HT, and DM during the follow-up period was not collected. In addition, incident comorbidities during the follow-up period could have affected event rates [26]; however, only the baseline clinical characteristics were used for the analysis in the present study.

## Conclusions

This sub-analysis showed that some components of the HAS-BLED score were independently associated with major bleeding in Japanese NVAF patients receiving anticoagulation therapy by rivaroxaban. Although the ROC curve analysis did not show accurate predictability for thromboembolic and bleeding events, the scores are effective for evaluating risk of thromboembolism and bleeding in NVAF patients treated with rivaroxaban. Additionally, CrCl value of 30–49 mL/min was an independent predictor of major bleeding in patients receiving rivaroxaban. Only prior history of stroke emerged as an independent predictor of stroke/SE, but other components of CHA_2_DS_2_-VASc score did not so.

## Abbreviations

AF: Atrial fibrillation
BP: Blood pressure
BNP: Brain-type natriuretic peptide
CI: Confidence interval
CrCl: Creatinine clearance
DM: Diabetes mellitus
DOAC: Direct oral anticoagulants
DVT: Deep vein thromboembolism
CHF: Congestive heart failure
HR: Hazard ratio
HF: Heart failure
HT: Hypertension
ROC: Receiver operating characteristic
ISTH: International Society on Thrombosis and Haemostasis
MI: Myocardial infarction
NSAIDs: Non-steroidal anti-inflammatory drugs
NVAF: Non-valvular atrial fibrillation
NT-pro BNP: N-terminal fragment of the prohormone brain-type natriuretic peptide
PAD: Peripheral arterial disease
PAF: Paroxysmal atrial fibrillation
PE: Pulmonary embolism
SD: Standard deviation
SE: Systemic embolism
TIA: Transient ischemic attack
