# Correction to: Clinical risk factors of stroke and major bleeding in patients with non-valvular atrial fibrillation under rivaroxaban: the EXPAND Study sub-analysis

**DOI:** 10.1007/s00380-019-01479-x

**Published:** 2019-08-02

**Authors:** Ichiro Sakuma, Shinichiro Uchiyama, Hirotsugu Atarashi, Hiroshi Inoue, Takanari Kitazono, Takeshi Yamashita, Wataru Shimizu, Takanori Ikeda, Masahiro Kamouchi, Koichi Kaikita, Koji Fukuda, Hideki Origasa, Hiroaki Shimokawa

**Affiliations:** 1 Cardiovascular Medicine, Hokko Memorial Clinic, Sapporo, Hokkaido Japan; 2 Center for Brain and Cerebral Vessels, Sanno Hospital and Sanno Medical Center, International University of Health and Welfare, 8-5-35 Akasaka, Minato-ku, Tokyo, Japan; 3 Minamihachioji Hospital, 3-18-12, Koyasu-cho, Hachioji, Tokyo Japan; 4 Saiseikai Toyama Hospital, 33-1, Kusunoki, Toyama, Toyama Japan; 5 Department of Medicine and Clinical Science, Graduate School of Medical Sciences, Kyushu University, 3-1-1, Maidashi, Higashi-ku, Fukuoka, Fukuoka Japan; 6 Cardiovascular Institute Hospital, 3-2-19 Nishiazabu, Minato-Ku, Tokyo, Japan; 7 Department of Cardiovascular Medicine, Graduate School of Medicine, Nippon Medical School, 1-1-5, Bunkyo-ku, Sendagi, Tokyo Japan; 8 Department of Cardiovascular Medicine, Faculty of Medicine, Toho University, 5-21-16, Omorinishi, Ota-ku, Tokyo, Japan; 9 Department of Health Care Administration and Management, Center for Cohort Study, Kyushu University Graduate School of Medical Sciences, 3-1-1, Maidashi, Higashi-ku, Fukuoka, Fukuoka Japan; 10 Department of Cardiovascular Medicine, Graduate School of Medical Sciences, Kumamoto University, 2-39-1, Kurokami Chuo-ku, Kumamoto, Kumamoto Japan; 11 Division of Heart Rhythm, International University of Health and Welfare Hospital, International University of Health and Welfare, 537-3, Iguchi, Nasushiobara, Tochigi Japan; 12 Division of Biostatistics and Clinical Epidemiology, University of Toyama Graduate School of Medicine, 2630 Sugitani, Toyama, Toyama Japan; 13 Department of Cardiovascular Medicine, Tohoku University Graduate School of Medicine, 1-1, Seiryomachi, Aoba-ku, Sendai, 980-8574 Miyagi Japan

## Correction to: Heart and Vessels 10.1007/s00380-019-01425-x

In the original publication of the article, the Figure 2b and the Tables 2 and 3 were published incorrectly.

The corrected Figure 2b and Tables 2 and 3 are provided below. 
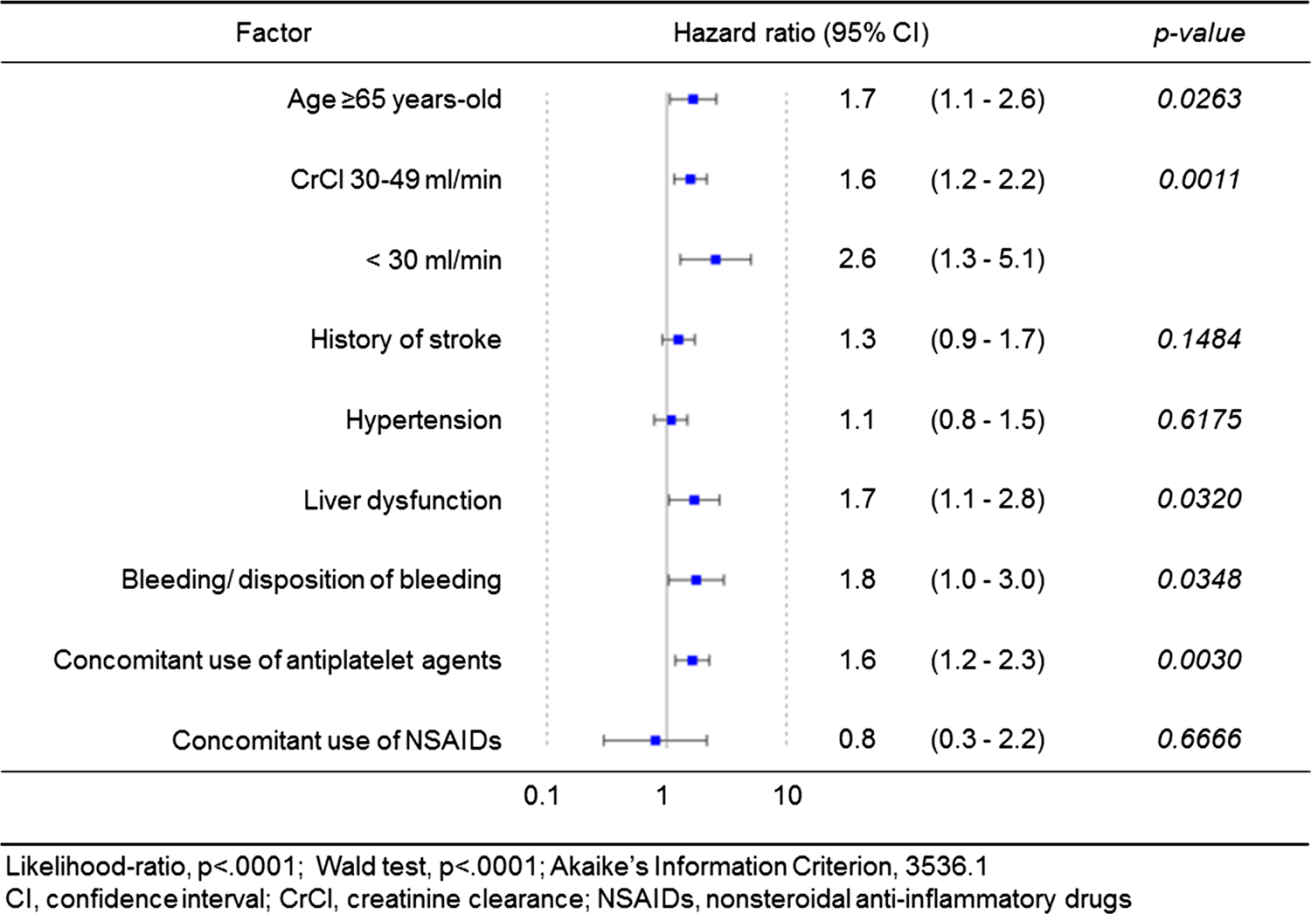

**Table 2:** Incidence rate and univariate analysis by Cox proportional hazards analysis of stroke/systemic embolism

	No. of event (%/year)	HR	95% CI	*p* value
Overall	176 (1.0)			
Sex				
Male	115 (1.0)	Reference		*0.5283*
Female	61 (1.1)	1.1	0.8, 1.5	
Age class 1 (years-old)				
< 65	23 (0.7)	Reference		*0.0176*
≥ 65	153 (1.1)	1.7	1.1, 2.6	
Age class 2 (years-old)				
< 65	23 (0.7)	Reference		*0.0022*
65–74	60 (0.9)	1.3	0.8, 2.2	
≥ 75	93 (1.3)	2.0	1.3, 3.2	
Body weight (kg)				
≥ 60	84 (0.8)	Reference		*0.0250*
50–59	50 (1.1)	1.3	0.9, 1.9	
< 50	33 (1.4)	1.7	1.1, 2.6	
Systolic blood pressure (mmHg)				
< 160	156 (1.0)	Reference		*0.0928*
≥ 160	12 (1.6)	1.6	0.9, 3.0	
CrCl (mL/min)				
≥ 50	112 (0.9)	Reference		*0.0026*
30–49	49 (1.5)	1.8	1.3, 2.5	
< 30	3 (1.0)	1.2	0.4, 3.7	
Comorbidity^a^				
Congestive heart failure				
–	129 (1.0)	Reference		*0.8550*
+	47 (1.0)	1.0	0.7, 1.4	
Hypertension				
–	44 (0.9)	Reference		*0.2768*
+	132 (1.1)	1.2	0.9, 1.7	
Angina pectoris				
–	157 (1.0)	Reference		*0.7193*
+	19 (0.9)	0.9	0.6, 1.5	
Diabetes mellitus				
–	121 (0.9)	Reference		*0.0227*
+	55 (1.3)	1.4	1.1, 2.0	
Aortic aneurysm				
–	174 (1.0)	Reference		*0.7916*
+	2 (0.8)	0.8	0.2, 3.3	
Deep vein thrombosis				
–	175 (1.0)	Reference		*0.9718*
+	1 (1.0)	1.0	0.2, 7.4	
Pulmonary embolism				
–	175 (1.0)	Reference		*0.4012*
+	1 (2.2)	2.3	0.3, 16.2	
Dyslipidemia				
–	105 (1.0)	Reference		*0.6278*
+	71 (1.0)	0.9	0.7, 1.3	
Liver dysfunction				
–	167 (1.0)	Reference		*0.6999*
+	9 (0.9)	0.9	0.5, 1.7	
Renal dysfunction				
–	176 (1.0)	Reference		*0.6995*
+	0 (0.0)	< 0.001	< 0.001	
Medical history^a^				
Stroke (ischemic/hemorrhagic)				
–	91 (0.7)	Reference		< *0.0001*
+	85 (2.3)	3.6	2.7, 4.8	
Transient ischemic attack				
–	169 (0.9)	Reference		*0.4721*
+	7 (1.3)	1.3	0.6, 2.8	
Systemic embolism				
–	174 (1.0)	Reference		*0.6643*
+	2 (1.4)	1.4	0.3, 5.5	
Vascular disease (MI/PAD)				
–	158 (1.0)	Reference		*0.0352*
+	18 (1.6)	1.7	1.0, 2.7	
Malignant tumor				
–	159 (1.0)	Reference		*0.7657*
+	17 (1.1)	1.1	0.7, 1.8	
Bleeding/disposition of bleeding				
–	169 (1.0)	Reference		*0.9900*
+	7 (1.0)	1.0	0.5, 2.1	
Rivaroxaban dosage				
15 mg/day	87 (0.9)	Reference		*0.0658*
10 mg/day	89 (1.2)	1.3	1.0, 1.8	
Amount of drinking (unit/week)				
No	105 (1.1)	Reference		*0.1202*
< 8	47 (0.8)	0.7	0.5, 1.0	
≥ 8	24 (1.0)	0.9	0.6, 1.4	
History of smoking				
No	99 (0.9)	Reference		*0.2940*
In the past	54 (1.1)	1.1	0.8, 1.6	
Current	23 (1.3)	1.4	0.9, 2.2	
Type of AF				
PAF	67 (0.9)	Reference		*0.0799*
Non-PAF^b^	109 (1.1)	1.3	1.0, 1.8	
Using concomitant anti-platelets^a^				
–	138 (0.9)	Reference		*0.0046*
+	38 (1.5)	1.7	1.2, 2.4	
Using concomitant NSAIDs^a^				
–	172 (1.0)	Reference		*0.9525*
+	4 (1.0)	1.0	0.4, 2.8	
CHADS_2_ score				
< 3	76 (0.7)	Reference		< *0.0001*
≥ 3	100 (1.7)	2.7	2.0, 3.6	
CHA_2_DS_2_-VASc score				
< 4	58 (0.6)	Reference		< *0.0001*
≥ 4	118 (1.5)	2.5	1.9, 3.5	
HAS-BLED score				
< 2	60 (0.6)	Reference		< *0.0001*
≥ 2	106 (1.7)	2.8	2.0, 3.8	

*HR* hazard ratio, *CI* confidence interval, *CrCl* creatinine clearance, *MI* myocardial infraction, *PAD* peripheral arterial disease, *AF* atrial fibrillation, *PAF* paroxysmal atrial fibrillation, *NSAIDs* non-steroidal anti- inflammatory drugs

^a^Reference; without factor

^b^Persistent and permanent atrial fibrillation

**Table 3:** Incidence rate and univariate analysis by Cox proportional hazards analysis of ISTH major bleeding

	ISTH major bleeding			
	No. of event (%/year)	HR	95% CI	*p* value
Overall	215 (1.2)			
Sex				
Male	147 (1.2)	Reference		*0.8223*
Female	68 (1.2)	1.0	0.7, 1.3	
Age class 1 (years-old)				
< 65	26 (0.7)	Reference		*0.0027*
≥ 65	189 (1.4)	1.9	1.2, 2.8	
Age class 2 (years-old)				
< 65	26 (0.7)	Reference		< *0.0001*
65–74	66 (1.0)	1.3	0.8, 2.1	
≥ 75	123 (1.7)	2.4	1.6, 3.6	
Body weight (kg)				
≥ 60	125 (1.3)	Reference		*0.7249*
50–59	52 (1.1)	0.9	0.7, 1.3	
< 50	31 (1.3)	1.1	0.7, 1.6	
Systolic blood pressure (mmHg)				
< 160	199 (1.3)	Reference		*0.2772*
≥ 160	6 (0.8)	0.6	0.3, 1.4	
CrCl (mL/min)				
≥ 50	138 (1.1)	Reference		< *0.0001*
30–49	59 (1.8)	1.8	1.3, 2.4	
< 30	9 (2.9)	2.9	1.5, 5.6	
Comorbidity^a^				
CHF				
–	149 (1.2)	Reference		*0.1140*
+	66 (1.4)	1.3	1.0, 1.7	
Hypertension				
–	53 (1.1)	Reference		*0.1770*
+	162 (1.3)	1.2	0.9, 1.7	
Angina pectoris				
–	181 (1.2)	Reference		*0.0543*
+	34 (1.7)	1.4	0.9, 2.1	
Diabetes mellitus				
–	159 (1.2)	Reference		*0.4925*
+	56 (1.3)	1.1	0.8, 1.5	
Aortic aneurysm				
–	210 (1.2)	Reference		*0.2144*
+	5 (2.1)	1.7	0.7, 4.2	
DVT				
–	212 (1.2)	Reference		*0.0823*
+	3 (3.1)	2.6	0.9, 8.3	
PE				
–	214 (1.2)	Reference		*0.5833*
+	1 (2.2)	1.7	0.2, 12.3	
Dyslipidemia				
–	125 (1.2)	Reference		*0.9155*
+	90 (1.2)	1.0	0.8, 1.3	
Liver dysfunction				
–	195 (1.2)	Reference		*0.0252*
+	20 (2.0)	1.7	1.1, 2.7	
Renal dysfunction				
–	215 (1.2)	Reference		*0.6682*
+	0 (0.0)	< 0.001	< 0.001, > 999.9	
Medical history				
Stroke (ischemic/hemorrhagic)				
–	154 (1.1)	Reference		*0.0065*
+	61 (1.7)	1.5	1.1, 2.0	
Transient ischemic attack				
–	208 (1.2)	Reference		*0.8415*
+	7 (1.3)	1.1	0.5, 2.3	
Systemic embolism				
–	215 (1.2)	Reference		*0.1736*
+	0 (0.0)	< 0.001	< 0.001, > 999.9	
Vascular disease (MI/PAD)				
–	196 (1.2)	Reference		*0.1345*
+	19 (1.7)	1.4	0.9, 2.3	
Malignant tumor				
–	189 (1.2)	Reference		*0.1145*
+	26 (1.7)	1.4	0.9, 2.1	
Bleeding/disposition of bleeding				
–	200 (1.2)	Reference		*0.0240*
+	15 (2.1)	1.8	1.1, 3.1	
Rivaroxaban dosage				
15 mg/day	108 (1.1)	Reference		*0.0635*
10 mg/day	107 (1.4)	1.3	1.0, 1.7	
Amount of drinking (unit/week)				
No	128 (1.4)	Reference		*0.0649*
< 8	57 (1.0)	0.7	0.5, 1.0	
≥ 8	30 (1.3)	0.9	0.6, 1.4	
History of smoking				
No	126 (1.2)	Reference		*0.4144*
In the past	71 (1.4)	1.2	0.9, 1.6	
Current	18 (1.0)	0.9	0.5, 1.4	
Type of AF				
PAF	89 (1.1)	Reference		*0.3453*
Non-PAF^b^	126 (1.3)	1.1	0.9, 1.5	
Using concomitant anti-platelets^a^				
–	166 (1.1)	Reference		*0.0003*
+	49 (2.0)	1.8	1.3, 2.5	
Using concomitant NSAIDs^a^				
–	211 (1.2)	Reference		*0.7265*
+	4 (1.0)	0.8	0.3, 2.3	
CHADS_2_ score				
< 3	121 (1.0)	Reference		*0.0009*
≥ 3	94 (1.6)	1.6	1.2, 2.1	
CHA_2_DS_2_-VASc score				
< 4	92 (1.0)	Reference		*0.0001*
≥ 4	123 (1.6)	1.7	1.3, 2.2	
HAS-BLED score				
< 2	92 (0.9)	Reference		< *0.0001*
≥ 2	111 (1.7)	1.9	1.4, 2.5	

*HR* hazard ratio, *CI* confidence interval, *ISTH* International Society on Thrombosis and Haemostasis, *CrCl* creatinine clearance, *MI* myocardial infraction, *PAD* peripheral arterial disease, *AF* atrial fibrillation, *PAF* paroxysmal atrial fibrillation, *NSAIDs* non-steroidal anti-inflammatory drugs

^a^Reference; without factor

^b^Persistent and permanent atrial fibrillation

